# Iron Content and Nutritional Quality of Commercial Complementary Baby Foods Available in Australia

**DOI:** 10.1155/jnme/9518894

**Published:** 2026-08-30

**Authors:** Ghazal Akbaridoust, Talicia Sutherland, Jimmy Chun Yu Louie

**Affiliations:** ^1^ Department of Allied Health, School of Health Sciences, Swinburne University of Technology, Hawthorn, Victoria, Australia, swinburne.edu.au

**Keywords:** baby food, complementary food, infant feeding, iron

## Abstract

**Background:**

Optimal nutrition in infancy supports growth, development and long‐term health. Commercial complementary foods are widely used in Australia, yet concerns persist regarding their iron, sugar and sodium content. This study examines their nutritional composition.

**Methods:**

In 2024, nutrition information was collected for complementary foods labelled from 6 months of age across four major retailers in Melbourne. Products for infants under 6 months and dairy‐only products were excluded. Nutrient composition was analysed for iron, energy, fat, protein, carbohydrate, total sugar and sodium. Added sugars and free sugars were identified from ingredient lists.

**Results:**

A total of 266 products were identified. Snacks had the highest average energy (1692.7 ± 257.3 kJ/100 g), protein (7.8 ± 4.6 g/100 g), fat (9.1 ± 7.5 g/100 g), carbohydrates (69.4 ± 12.7 g/100 g), sodium (82.1 ± 109.9 mg/100 g) and iron (8.2 ± 10.9 mg/100 g), with some products containing up to 38 mg/100 g. Only 19.2% of all products were iron‐fortified, and 10 of these products did not declare iron on the label. Iron‐fortification was limited to grain‐based products, most commonly breakfast cereals (58.8%) and snacks (37.3%). No fruit purees or main meals were iron‐fortified. Fruit purees had high sugar content (9.7 g/100 g). Free sugars were present in 24.1% of products, most commonly snacks (46.4%).

**Conclusions:**

Most commercial complementary foods provided little iron, and fortified products did not consistently declare iron content. Snacks were the most energy‐, sugar‐ and sodium‐dense category. Stronger standards and clearer labelling may better support infant feeding.

## 1. Introduction

Feeding practices in the first 36 months of life, spanning infancy (0–12 months) and toddlerhood (12–36 months), influence nutrition, growth, development and long‐term food preferences and dietary patterns [1, 2]. During this period, infants undergo growth and neurodevelopment that increase their requirements for energy and nutrients [3]. Nutrient‐poor dietary patterns established during infancy can affect health and development in ways that endure later on in life [1, 2].

The World Health Organization (WHO) [4] and Australian Infant Feeding Guidelines [5] recommend that from the age of 6 months, infants should start receiving complementary foods (foods and drinks other than breastmilk or infant formula) to provide essential nutrients, such as iron. WHO suggests that during this phase of life, infants should limit their intake of free sugars to 5%–10% of their overall energy intake and limit their consumption of foods that contain additional sugars or sodium. Additionally, no sugar should be added to foods before 1 year of age [4]. With childhood obesity increasing globally and early rapid weight gain linked to higher obesity risk later in life [6], the nutrition quality of food, especially sugar and sodium in commercial complementary food, during early childhood is becoming more crucial [7].

Commercial complementary foods are widely available in Australia and are often purchased by caregivers of infants for their convenience and perceived value, although evidence indicates that they are not economical relative to home‐prepared foods [8]. The market is valued at approximately AU$1.2 billion, and squeeze pouches have become the most dominant format, rising from 28% of sales in 2012 to 43% in 2022 [9, 10]. Internationally, in the EU Childhood Obesity Project cohort, 68% of children were still fed commercial complementary foods at 24 months of age, and in Spain, Italy and Poland over 95% of infants and children who were fed commercial complementary foods at 9 and 12 months consumed at least one sweetened commercial complementary food [11].

Iron deficiency is among the most common nutritional deficiencies worldwide, and infants are especially vulnerable because of their growth rate and limited iron stores after 6 months [12–14]. Breastmilk alone cannot meet iron needs beyond this age [15], and many complementary foods are naturally low in bioavailable iron [16]. Without fortified options, meeting daily requirements can be difficult [17, 18]. Whole and family foods are the recommended means of meeting iron needs during complementary feeding [5]. For families who nonetheless use commercial products, current labelling offers limited support: Iron content need only be declared when a product carries an iron‐related claim [19]. Because many caregivers do not consult or readily interpret the nutrition information panel [20, 21], voluntary, claim‐triggered disclosure is an unreliable basis for informed choice. Studies show that few pouch products in Australia report iron content or are fortified, putting infants relying on these foods at risk [9, 10, 22]. This pattern is consistent with concerns raised elsewhere that many pouch products are predominantly pureed fruit, and fruit‐only pouch meals do not supply appreciable amounts of key nutrients that need to be provided during complementary feeding, particularly iron [23].

International research reinforces concerns about the nutritional quality of commercial infant foods, particularly regarding iron content. A review of products sold in Hong Kong found that less than 20% were fortified with iron, and only a small number listed iron content [24]. Similarly, a 2020 Australian audit reported that just 12% of products disclosed iron levels, and many were high in free sugars [9], while subsequent analyses have found that most squeeze‐pouch products contain free sugars, primarily from fruit purees and juice concentrates, while only a small percentage meet WHO benchmarks for nutrient composition [10, 22, 25].

Beyond iron, sugar and sodium exceeding the WHO nutrient and promotion profile model thresholds for foods for infants and young children [26] are also recurring issues in commercial infant foods. Global audits have identified that many of these products contain added sugars, high sodium levels and other ingredients that may not support healthy early feeding [8, 9, 18, 24, 27–30]. Recent Australian audits report that most squeeze‐pouch products contain free sugars and that few meet WHO nutrient benchmarks [8, 10]. Internationally, a large US study of over 1,000 products found that toddler‐targeted items frequently exceeded sodium and added‐sugar limits [29], a pattern with potential relevance to Australia’s growing commercial infant‐food market. This study provides updated data on the nutrient content, particularly iron, of complementary foods sold by major Australian retailers and contributes to a growing body of evidence calling for stronger standards and clearer labelling.

## 2. Methods

This study used a cross‐sectional audit design to assess commercial complementary infant foods available in the Australian market. Data were collected in 2024. Products were sampled from four major retailers, namely, Aldi, Coles, Woolworths and Chemist Warehouse, across two Melbourne suburbs (Broadmeadows and Camberwell) representing different socioeconomic areas (classified using the ABS Socio‐Economic Indexes for Areas [SEIFA]). Eligible products were commercial complementary foods labelled for infants and young children from 6 months of age, spanning breakfast cereals, fruit purées, main meals and snacks. The dedicated infant/baby‐food aisle was searched in each store. Broadmeadows (a lower‐SEIFA area) and Camberwell (a higher‐SEIFA area) were selected because both contained the full set of target retailers, enabling the same chains to be sampled across contrasting socioeconomic catchments. It is acknowledged that, given the limited sampling areas, findings may not fully capture all available products nationally.

Two researchers independently photographed the front and back of each product’s packaging capturing brand names, health claims, target age groups, ingredient lists and nutrition information. Products intended for infants under 6 months and dairy‐only products (e.g., flavoured milk, yogurt, cheese and custard) were excluded. Dairy‐only products fall outside the complementary‐food categories under audit and are governed by separate compositional standards [31]; fruit purées were retained because they are marketed and used as complementary first foods. Photographic records were cross‐checked against retailer and manufacturer websites to confirm accuracy and completeness. Products were catalogued once and deduplicated across sites; therefore, a product recorded at one suburb may also have been stocked at the other. The sampling approach aimed to reflect the range of complementary infant foods available in the Australian market. Similar to the strategy used by Cheung et al. [24], our approach ensured that a wide array of products was evaluated, although differences in regional labelling practices were noted.

### 2.1. Product Classification, Data Entry and Data Analysis

Products were categorised into four main groups: breakfast cereals, fruit purees, main meals and snacks. Nutritional data including energy, carbohydrate, sugars, protein, fat, saturated fat, iron and salt content were recorded per 100 g, with iron additionally recorded per serve. For breakfast cereals, values were recorded for the product as sold (dry) because preparation liquids and reconstitution ratios vary across products and households and are not standardised on‐pack. Any values reported as ‘less than’ a threshold (e.g., ‘less than 0.5 g’) was recorded as half that threshold (0.25 g) to enable the calculation of descriptive statistics. The target age group was recorded as the youngest age group that the product was targeting. Packaging text matching the regulatory definition of a nutrition content or health claim [19] was recorded as the presence of a claim. The number of claims per product was also recorded. Added sugars were defined as sugars and syrups added as discrete ingredients, including sugar, cane sugar, dextrose, glucose, maltodextrin and honey, identified from ingredient lists [32]. Free sugars comprised added sugars together with sugars from fruit juice and fruit juice concentrate, consistent with the WHO definition; sugars intrinsic to whole‐fruit purees were therefore not counted as free sugars [33]. Products were classified as iron‐fortified where an iron compound (e.g., mineral iron and ferrous fumarate) appeared in the ingredient list. For products where iron content was not reported on the nutrition panel, we calculated iron content based on the percentage of iron‐rich ingredients, defined as containing > 2 mg/100 g of iron in the Australian Food Composition Database [34], including meat (beef and lamb), poultry (chicken), fish, iron‐rich vegetables, legumes and cereals (oats and quinoa). Declared ingredient percentages were used to calculate the proportional iron content where available; otherwise, iron content was estimated from ingredient order and typical compositions, following approaches used in previous complementary‐food audits [10, 24]. These estimates are approximate and were interpreted cautiously. For iron fortificants without percentage declarations, we used 0.1% by weight as a conservative estimate. Descriptive statistics were generated for each product category using R (Version 4.4.1).

## 3. Results

### 3.1. Sample and Product Characteristics

Our audit identified 266 unique commercial complementary infant food products from the included retail chains. Snack‐based products comprised the largest portion at 41.4% (*n* = 110), followed by main meals at 28.6% (*n* = 76), fruit purees at 23.7% (*n* = 63) and breakfast cereals at 6.4% (*n* = 17). Main meals had the largest average serving size (144.5 ± 33.4 g), followed by fruit purees (115.5 ± 9.7 g), breakfast cereals (89.5 ± 53.2 g) and snacks (15.7 ± 21.9 g). Breakfast cereals were the most expensive per unit (AU$4.3 ± 2.1), with snacks (AU$3.3 ± 1.5), main meals (AU$2.2 ± 0.8) and fruit purees (AU$1.9 ± 0.6) following (Table 1).

**TABLE 1:** Serving size, unit price and content of selected nutrients of the sampled products (*n* = 266).

Variable	*n*	Mean	SD	Min	Q1	Median	Q3	Max	IQR
*Serving size* (*g*)									
Breakfast cereals	17	89.5	53.2	8	20	111	120	150	100
Fruit purees	63	115.5	9.7	85	120	120	120	120	0
Main meals	76	144.5	33.4	50	120	120	170	220	50
Snacks	110	15.7	21.9	3.2	8	12	15	125	7
Overall	266	80.8	62.6	3.2	12	120	120	220	108

*Unit price (AU$)*									
Breakfast cereals	17	4.3	2.1	1.0	2.5	4.4	6	6.9	3.5
Fruit purees	63	1.9	0.6	1.0	1.6	2	2.2	2.9	0.6
Main meals	76	2.2	0.8	1.0	1.8	2.2	3	4.9	1.2
Snacks	110	3.3	1.5	1.0	2	3.1	4.5	7	2.5
Overall	266	2.8	1.4	1.0	1.8	2.2	3.4	7	1.6

*Energy per 100 * *g* *(kJ)*									
Breakfast cereals	17	484.3	501.4	170	240	295	317	1609	77
Fruit purees	63	294.7	51.4	192	252.5	285	338	400	85.5
Main meals	76	267.3	155.7	176	224.5	247.5	274.5	1560	50
Snacks	110	1692.7	257.3	336	1562.5	1700	1800	2400	237.5
Overall	266	877.1	723.3	170	252.3	334.5	1661.8	2400	1409.5

*Protein per 100 * *g*(*g*)									
Breakfast cereals	17	1.7	0.4	1	1.3	1.9	2.1	2.4	0.8
Fruit purees	63	1.1	0.6	0.2	0.7	1.1	1.4	3.1	0.7
Main meals	76	2.9	1.2	1	2.2	2.9	3.2	11.3	1.0
Snacks	110	7.8	4.6	0.5	5.5	6.9	8.0	24.1	2.45
Overall	266	4.4	4.2	0.2	1.5	2.9	6.4	24.1	4.9

*Total fat per 100 * *g*(*g*)									
Breakfast cereals	17	2.1	3.3	0.2	0.7	0.9	1.2	10.9	0.5
Fruit purees	63	0.8	0.6	0.1	0.3	0.5	1.2	3.1	0.9
Main meals	76	1.2	0.7	0.2	0.5	1	1.6	2.9	1.1
Snacks	110	9.1	7.5	0.1	1.3	7.8	14.8	31.5	13.5
Overall	266	4.4	6.3	0.1	0.5	1.2	6.2	31.5	5.7

*SFA per 100 * *g*(*g*)									
Breakfast cereals	6	0.8	0.8	0.1	0.2	0.7	1.2	2.2	1.0
Fruit purees	44	0.5	0.4	0	0.1	0.5	0.6	1.5	0.5
Main meals	61	0.5	0.5	0	0.1	0.3	0.6	1.9	0.5
Snacks	102	2.3	3.4	0.1	0.5	1.4	2.4	18.7	1.9
Overall	213	1.4	2.5	0	0.2	0.6	1.4	18.7	1.2

*CHO per 100 * *g*(*g*)									
Breakfast cereals	17	21.0	22.1	7.6	9.5	11.9	16.3	83	6.8
Fruit purees	63	14.0	2.5	9.3	12	13.6	15.8	20.5	3.8
Main meals	76	9.7	7.7	5	7.7	8.7	10	74.4	2.3
Snacks	110	69.4	12.7	13.1	62.3	68.3	75.8	92.8	13.5
Overall	266	36.1	30.1	5	10.2	15.4	65.5	92.8	55.3

*Total sugar per 100 * *g* *(g)*									
Breakfast cereals	17	5.3	6.3	0.1	1	4.2	5.1	23.1	4.1
Fruit purees	63	9.7	3.1	3.9	7.45	8.9	11.7	16.5	4.2
Main meals	76	2.6	1.2	0.8	1.8	2.7	3.0	7.6	1.2
Snacks	110	19.8	20.1	0.6	4.1	10	30.5	68.8	26.4
Overall	266	11.6	15.1	0.1	2.9	6.6	12.2	68.8	9.3

*Sodium per 100 * *g* *(mg)*									
Breakfast cereals	17	45.4	101.4	0.25	2.5	5	17	327	14.5
Fruit purees	63	7.3	5.4	1.0	2.8	7	11	26	8.3
Main meals	76	19.8	15.1	2.5	10	15	23	73	13
Snacks	110	82.1	109.9	0	6	26.5	144.8	409	138.8
Overall	266	44.2	82.2	0	5	13	29.7	409	24.7

^∗^ *Iron (label) per 100 * *g* *(mg)*									
Breakfast cereals	10	3.8	1.4	2	2.5	3.7	5.1	5.6	2.6
Fruit purees	0	—	—	—	—	—	—	—	—
Main meals	0	—	—	—	—	—	—	—	—
Snacks	31	21.2	8.3	2.4	20	20	24.2	38	4.2
Overall	41	16.9	10.5	2	5	20	23	38	18

^∗^ *Iron (label) per serve (mg)*									
Breakfast cereals	10	4.4	1.3	2.3	3.3	4.7	5.0	6.2	1.7
Fruit purees	0	—	—	—	—	—	—	—	—
Main meals	0	—	—	—	—	—	—	—	—
Snacks	31	3.3	3.6	0.8	1.6	2.4	2.8	20	1.2
Overall	41	3.6	3.2	0.8	2	2.6	4.8	20	2.8

^#^ *Iron per 100 * *g* *(* *m* *g*)									
Breakfast cereals	17	2.5	1.9	0	0.7	2.5	3.7	5.6	3.0
Fruit purees	63	0.1	0.2	0	0	0.0	0.1	1.1	0.1
Main meals	76	0.2	0.1	0	0.1	0.1	0.2	0.7	0.2
Snacks	110	8.2	10.9	0	0	1.5	20	38	20
Overall	266	3.6	8.0	0	0	0.1	1.4	38	1.4

^#^ *Iron per serve (* *m* *g* *)*									
Breakfast cereals	17	3.0	2.3	0	0.6	3.1	5.0	6.5	4.4
Fruit purees	63	0.1	0.2	0	0	0.0	0.2	0.6	0.2
Main meals	76	0.2	0.2	0	0.1	0.2	0.4	0.9	0.3
Snacks	110	1.0	2.3	0	0	0.1	1.3	20	1.3
Overall	266	0.7	1.8	0	0	0.1	0.5	20	0.5

*Note:* CHO, carbohydrates; SFA, saturated fat.

^∗^Iron content taken from the nutrition information panel.

^#^Iron content estimated based on the proportion of high‐iron ingredients or, where available, taken from the nutrition information panel. Where no high‐iron ingredients were identified, iron content was assumed to be negligible and coded as 0 mg.

### 3.2. Macronutrients and Energy

Snacks had the highest energy density (1692.7 ± 257.3 kJ/100 g), protein (7.8 ± 4.6 g/100 g), fat (9.1 ± 7.5 g/100 g), carbohydrate (69.4 ± 12.7 g/100 g) and total sugar content (19.8 ± 20.1 g/100 g). Fruit purees were the second highest in sugar (9.7 ± 3.1 g/100 g), while breakfast cereals (5.3 ± 6.3 g/100 g) and main meals (2.6 ± 1.2 g/100 g) contained less. Protein content was the next highest in main meals (2.9 ± 1.2 g/100 g), followed by breakfast cereals (1.7 ± 0.4 g/100 g) and fruit purees (1.1 ± 0.6 g/100 g) (Table 1).

### 3.3. Sodium

Sodium levels ranged from 0 to 409 mg/100 g, with snacks having the highest average (82.1 ± 109.9 mg/100 g), ahead of breakfast cereals (45.4 ± 101.4 mg/100 g), main meals (19.8 ± 15.1 mg/100 g) and fruit purees (7.3 ± 5.4 mg/100 g) (Table 1).

### 3.4. Health Claims

**Figure 1:**
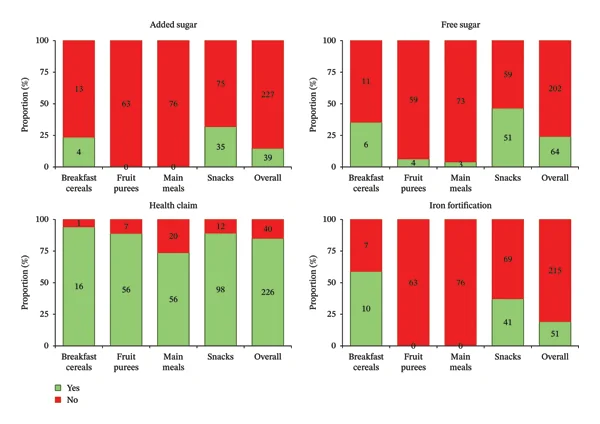
Presence of added sugar, free sugar, health claims and iron fortification in the sampled products. Bars show the number of products meeting each criterion (green) versus those that do not (red), with counts displayed within the bars.

Health claims were near‐ubiquitous, appearing on 85.0% (*n* = 226) of all products, more than four times the proportion that was iron‐fortified (Figure 1).

### 3.5. Iron Content and Fortification

Iron fortification was present in 19.2% (*n* = 51) of products. Fortification was confined to grain‐based categories and was most frequent in breakfast cereals (58.8% of breakfast cereals, *n* = 10), followed by snacks (37.3%, *n* = 41); no fruit puree or main meal was fortified (Figure 1). Snacks had the highest iron content per 100 g (8.2 ± 10.9 mg), with some products containing up to 38 mg, whereas breakfast cereals delivered the most iron per serving (3.0 ± 2.3 mg), reflecting their larger serving size. Ten of the 51 fortified products did not report iron levels on their nutrition panels (Table 1).

### 3.6. Added and Free Sugars

Added sugar was listed on 14.7% (*n* = 39) of products, confined to snacks (*n* = 35) and breakfast cereals (*n* = 4); all fruit purees (*n* = 63) and main meals (*n* = 76) were free of added sugar. Free sugar appeared in 24.1% (*n* = 64), most commonly in snacks (46.4%, *n* = 51) and breakfast cereals (35.3%, *n* = 6) and less often in fruit purees (6.3%, *n* = 4) and main meals (3.9%, *n* = 3) (Figure 1).

## 4. Discussion

This audit of commercial complementary infant foods in Australia identifies notable gaps in iron content and fortification. Most products lacked iron fortification and provided negligible iron levels, falling short of what infants need for healthy growth and development. These findings match international patterns and point to a clear disconnect between what these foods offer and what infants require.

Our analysis shows that 80.8% of the surveyed products were not fortified with iron. This gap is especially relevant for families relying heavily on fruit puree products and pouches, which commonly provide fruit‐only meals that do not supply appreciable amounts of iron needed during complementary feeding [23]. Where fortification was present, it clustered in grain‐based products and was proportionally most common in breakfast cereals, yet cereals were also the smallest category, representing just 6.4% of products. Fortification and labelling were inconsistent even within this group [11, 23, 35]. In practice, caregivers and product marketing often treat a product’s category, for example, that an item is a ‘main meal’ or a ‘baby’ pouch, as an implicit signal of nutritional adequacy [10, 21]. Our findings show this signal to be unreliable for iron: Category did not predict iron content or fortification in the absence of on‐pack micronutrient reporting. This is relevant for main meals, which are positioned as substantive components of an infant’s diet yet, in our sample, provided negligible iron [10].

This concern is grounded in national data: Approximately three‐quarters of Australian infants aged 6–12 months consume less iron than the estimated average requirement [18]. Low fortification would be reassuring only if these infants were meeting iron needs from other sources, which population intake data indicate many are not. International audits of commercial complementary cereals [24, 29] also report that only a minority contain iron and other priority micronutrients, reinforcing the need for clearer standards and labelling if such products are expected to help meet requirements. The results here reinforce those concerns within the Australian market [10]. We also found that 10 of the 51 products with added iron did not report iron content on the nutrition panel. This lack of labelling consistency deserves closer regulatory attention. Consistent with the Australian Infant Feeding Guidelines and Australian Dietary Guidelines, iron‐rich whole foods, such as meat, poultry, fish, legumes, iron‐rich vegetables and iron‐fortified infant cereal, are the recommended means of meeting these needs [5, 18]. For the subset of infants fed predominantly commercial products, the current low‐fortification landscape compounds the shortfall.

The categories differed in ways that resist a simple ranking. Main meals were lowest in sugar and sodium but negligible in iron; fruit purées had no added sugar but high free sugar and no fortification; snacks were also the highest in protein (7.8 vs. 2.9 g/100 g for main meals), a difference that likely reflects the dry‐weight, grain‐ and legume‐based formats of puffs and biscuits rather than superior nutritional value.

While most products met the WHO nutrient and promotion profile model benchmark [26] for added sugars, commercial complementary foods as a category raised concerns for energy, sugar and sodium content, with snacks being the most energy‐dense subcategory. Snacks comprised 41.4% of all surveyed products and averaged 1692.7 kJ/100 g. Audits of the broader commercial infant food market support these concerns: One study found that sugar levels ranged from 0.8 to 17.5 g/100 g, with 73% of products containing free sugars, mostly fruit‐based, even when labelled ‘no added sugar’ [10]; another reported that 78% of products failed to meet WHO nutritional benchmarks, particularly for energy density and sugar [22]; and only 28% met all WHO nutritional standards, with none meeting promotional guidelines [25].

Sodium was the highest in snacks, averaging 82.1 mg/100 g and reaching 409 mg/100 g, with several snack products exceeding the WHO sodium threshold for this age group; although average levels were lower than those reported in some international audits [28–30], elevated sodium exposure in the first two years carries implications for blood‐pressure trajectories and for the development of salt preference. Fruit purees, while free of added sugar, averaged 9.7 g of total sugar per 100 g, and heavy reliance on fruit‐based products may limit vegetable exposure and acceptance of less‐sweet flavours [9]. Taken together, these findings have direct implications for feeding practice: Caregivers who rely on commercial complementary foods may unintentionally leave iron gaps in their child’s diet and inadvertently expose infants to excess energy, sugar and sodium, and health professionals should emphasise iron‐rich whole foods and consider supplementation advice where indicated.

This study’s strengths include its broad coverage of 266 products from major retailers and the detailed focus on iron content. However, certain limitations must be acknowledged. The study was limited to products sampled in Melbourne and relied on estimated iron content for many items. Actual iron bioavailability was not assessed: nonhaem iron from fortificants and plant sources is absorbed far less efficiently than haem iron [36]. Furthermore, health claims were recorded as present/absent but not systematically categorised by claim type. These factors should be considered when interpreting the findings. The analysis focused on iron (and selected macronutrients and sodium) and did not assess other critical micronutrients relevant to infant and toddler nutrition (for example, zinc or iodine), which should be examined in future audits. Future work should also assess the contribution of commercial complementary foods to overall iron status in Australian infants and investigate how parental knowledge and purchasing decisions affect infant nutrition.

## 5. Conclusion

Most commercial complementary infant foods in Australia are not fortified with iron and provide little of it, while those that are fortified do not reliably declare it on the nutrition panel. Iron is concentrated in breakfast cereals, the smallest category, whereas snacks, the largest, are the most energy‐, sugar‐ and sodium‐dense. Whole and family foods remain the first‐line means of meeting infants’ iron and broader nutritional needs, and caregivers should be supported to prioritise them. For the commercial products that nonetheless form a substantial part of many infants’ diets, most lack iron fortification, free sugars were present in nearly a quarter of all products and in almost half of snacks, and sodium levels in some products exceeded WHO thresholds for this age group. As harm‐reduction measures, regulators should consider mandatory on‐pack iron declaration for complementary foods, clearer compositional standards for energy density and free‐sugar content in snacks and targeted iron fortification in the main‐meal and cereal categories positioned to contribute meaningfully to infants’ iron intake.

## Author Contributions

Ghazal Akbaridoust and Talicia Sutherland collected, analysed and interpreted the data and drafted the manuscript; Jimmy Chun Yu Louie conceived, designed and supervised the study, interpreted the data, contributed substantially to the discussion and critically reviewed the manuscript.

## Funding

This study was completed without external funding. Open access publishing facilitated by Swinburne University of Technology, as part of the Wiley ‐ Swinburne University of Technology agreement via the Council of Australasian University Librarians.

## Disclosure

All authors agree with the manuscript and declare that the content has not been published elsewhere.

## Conflicts of Interest

The authors declare no conflicts of interest.

## Data Availability

The data that support the findings of this study are available from the corresponding author upon reasonable request.
